# Functional annotation of novel heat stress-responsive genes in rice utilizing public transcriptomes and structurome

**DOI:** 10.1093/bioadv/vbag013

**Published:** 2026-01-21

**Authors:** Sora Yonezawa, Hidemasa Bono

**Affiliations:** Graduate School of Integrated Sciences for Life, Hiroshima University, Hiroshima 739-0046, Japan; Genome Editing Innovation Center, Hiroshima University, Hiroshima 739-0046, Japan

## Abstract

**Motivation:**

Life science databases include large collections of public transcriptome and large-scale structural data. The reuse and integration of these datasets may facilitate the identification of understudied genes and enable functional annotation across distantly related species, including plants and humans.

**Results:**

In this study, we used heat stress-responsive genes in rice as a model to functionally annotate previously understudied genes by integrating publicly available transcriptome data with structural information from the AlphaFold Protein Structure Database. Initially, we conducted a meta-analysis of public heat stress-related transcriptome datasets, identified gene groups, and verified stress-related terms through enrichment analysis. Subsequently, we performed structural alignment and sequence alignment between rice and human proteins, focusing on candidates exhibiting low sequence similarity but high structural similarity. We further incorporated supplemental data from public databases, including shared domain information between rice and human. This approach yielded a unique set of these candidates, notably those associated with metal homeostasis, such as iron and copper metabolism. Overall, our integrative method provided insights into these genes by leveraging diverse, publicly available datasets.

**Availability and implementation:**

The “plant2human workflow” for this analysis is available at https://doi.org/10.48546/WORKFLOWHUB.WORKFLOW.1206.10.

## 1 Introduction

Public databases in life sciences host diverse types of data and vast amounts of information. The National Genomics Data Center Database Commons, a comprehensive repository that curates life science databases, comprises over 7300 life science-related databases across 13 categories, including “Gene genome and annotation,” “Health and medicine,” and “Expression” (accessed 12 September 2025) (Ma *et al.* 2023). Access to these data resources supports the development of biological research and analyses.

Among these resources, transcriptome data have expanded rapidly due to the widespread adoption of next-generation sequencing. For example, the Gene Expression Omnibus (GEO) at the US National Center for Biotechnology Information (NCBI) is a public database that stores gene expression and epigenomic data; currently, the “Expression profiling by high throughput sequencing” category contains over 120 000 series (https://www.ncbi.nlm.nih.gov/geo/summary/? type=series) (accessed 13 September 2025) (Clough *et al.* 2024). NCBI GEO data can be reused for various purposes, including the integration of different datasets to identify new biological trends (Clough *et al.* 2024). We previously conducted a meta-analysis of human and mouse heat-stressed transcriptome data from NCBI GEO, integrating transcription factor binding and literature data. This analysis identified heat stress-related genes that are relatively understudied compared to heat shock proteins (HSPs) (Yonezawa and Bono 2023). This approach can be applied to plant research. For example, meta-analyses of publicly available RNA-sequencing (RNA-Seq) datasets have been conducted in *Arabidopsis thaliana* to identify stress-responsive genes under various stress conditions (Shintani *et al.* 2024). Hence, the reuse of public transcriptomic data, particularly through meta-analysis, facilitates new approaches to gene exploration.

As the volume of data accumulated in public databases has increased, new types of massive data have emerged. The AlphaFold Protein Structure Database (AFDB) has become a notable resource, containing more than 214 million predicted protein structures, including those of plants (Varadi *et al.* 2022, 2024). As a result, it has become feasible to investigate biological questions through the lens of the large-scale protein “structurome.” Studies using the AFDB have explored novel protein structure clusters and domain information, yielding novel biological insights (Barrio-Hernandez *et al.* 2023, Lau *et al.* 2024).

Quantitative analyses have shown that structural regions within the core regions of conserved protein domains are three to ten times more conserved than their sequences (Illergård *et al.* 2009), which broadens the range of genes available for comparative and functional annotation, including between distantly related species, such as plants and humans. Functional annotation of plant and human genes has been documented in several studies. In *Arabidopsis*, research has confirmed the function of genes similar to the human *BRCA2* (BRCA2 DNA repair associated) gene (Siaud *et al.* 2004). Furthermore, investigations into genes orthologous to human disease-related genes in *Arabidopsis* and *Populus trichocarpa* suggest the potential applicability of plant models in human disease research (Wang *et al.* 2023). However, approaches primarily based on sequence similarity may overlook genes with limited sequence conservation. Overall, the conservation of protein structure and the availability of large-scale structural data could expand the scope for identifying relationships among distantly related species, supporting further cross-species comparisons and annotations.

The expansion of public transcriptome data and the emergence of the structurome (AFDB) reflect the broadening scope of publicly available resources in the life sciences. Recently, the concept of “Unknome” has been proposed. The research group that proposed the Unknome identified clusters using the “knownness score,” a metric derived from Gene Ontology (GO) annotation evidence codes from the Universal Protein Resource (UniProt), and subsequently established the Unknome database. Genes within clusters with low knownness scores represent those that have received limited attention. Genes with low knownness scores that were conserved in humans and *Drosophila melanogaster* and present in at least 80% of the available metazoan genomes were selected and functionally validated via RNAi in *D. melanogaster*. These genes may be involved in essential functions, including stress resistance (Rocha *et al.* 2023). Therefore, leveraging public databases to identify understudied genes can facilitate their discovery and support further biological research.

In this study, public transcriptomes and structural data were integrated to develop an analytical workflow for identifying understudied genes and facilitating functional annotation. As a proof of concept, public rice (*Oryza sativa*) transcriptomes related to heat stress were mapped to the AFDB structure database. Rice serves as a suitable model organism for this purpose because of its comprehensive public transcriptome datasets, especially concerning heat stress. Additionally, HSPs, which play an important role of the rice heat stress response (Ren *et al.* 2023, Wang *et al.* 2024), are widely conserved at the protein sequence level across distantly related species, including humans; HSP90 is one of the representative example (Raman and Suguna 2015). Therefore, this approach was used to identify heat stress-responsive genes that, while lacking sequence similarity, share similar protein structures. By comparing these findings to human structural data, we identified understudied genes and conducted functional annotations based on existing human knowledge.

## 2 Methods

### 2.1 Curation of heat stress-related rice transcriptome data

We curated heat stress-related rice (*O. sativa*) RNA-Seq datasets from NCBI GEO (Clough *et al.* 2024) using the following search query: ((“heat stress” OR “heat shock” OR “heat shock response” OR “high temperature” OR “high temperature stress” OR “heat-sensitive” OR “heat stress tolerance” OR “high night temperature”) AND “Oryza sativa”[porgn:__txid4530]) AND “expression profiling by high throughput sequencing”[Filter] (accessed 20 January 2024). Data, including japonica and indica subspecies, were manually curated based on the described experimental conditions and complete data for heat stress and control conditions.

### 2.2 Transcriptome data processing and quantification

Fasterq-dump from the NCBI SRA Toolkit (ver. 3.0.1) (*Download SRA Sequences from Entrez Search Results*), fastp (ver. 0.23.4) (Chen 2023), and salmon (ver. 1.10.2) (Patro *et al.* 2017) were used for FASTQ file retrieval, trimming, and expression quantification. For expression quantification in salmon, the japonica subspecies transcript FASTA file was retrieved from Ensembl Plants release 58 (accessed 1 February 2024) (Yates *et al.* 2022) as a reference transcript. Gene expression levels were summarized using tximport software (Bioconductor package release ver. 3.19) (Soneson *et al.* 2015) and calculated as scaled transcripts per million (scaled TPM).

### 2.3 Calculation of expression ratio and HN-score

Following our previously described study protocol (Yonezawa and Bono 2023), we defined the Heat-stress and Non-treatment scores (HN-score) to capture the gene-level responses to heat stress. The calculation procedure is summarized below:

#### 2.3.1Calculation of expression ratio (HN-ratio)

The heat stress and non-treatment condition data were paired, and expression ratios (HN-ratio) were calculated for all genes using the following Equation (1):


(1) HN-ratio=THS+1Tnon-treatment+1  

where *T*_HS_ and *T*_non-treatment_ are the scaled TPM for each gene under heat stress and non-treatment condition pairs, respectively.

#### 2.3.2Gene classification based on HN-ratio and calculation of HN-score

Based on the HN-ratio and defined five-fold and 1/5-fold thresholds, all genes were classified as “upregulated,” “downregulated,” or “unchanged.” For instance, if the HN-ratio of Gene A in a given heat stress and non-treatment pair was 5, it was considered “upregulated,” while if the HN-ratio for Gene B was 1/5, it was considered “downregulated.” If Gene C did not meet either threshold, it was considered “unchanged.”

The counts in the three categories were aggregated across all the pairs. The HN-score was calculated as the difference between the “upregulated” and “downregulated” counts for each gene. The top and bottom genes were selected based on their HN-scores and used for subsequent analyses. Detailed annotation information for these selected genes was obtained from the Rice Annotation Project Database (version IRGSP-1.0 2025-03-19) (accessed 25 July 2025) (Sakai *et al.* 2013).

### 2.4 Enrichment analysis and semantic similarity analysis

Enrichment analysis was performed using GOAtools (ver. 1.4.12) (Klopfenstein *et al.* 2018) with GO annotation data obtained from Ensembl Plants release 58 on a selected group of rice genes. Obsolete GO terms were replaced using the GO release 2025-03-16 (Gene Ontology Consortium 2023). The semantic similarity among the enriched terms was calculated using simona (ver.1.4.0) (Gu 2024) with the “Sim_WP_1994” method (Wu and Palmer 1994). The similarities of all term pairs were clustered and visualized using the SimplifyEnrichment package (ver. 2.0.0) with the *binary cut* algorithm (“binary_cut”), which recursively performs divisive clustering to generate a dendrogram and then cuts it based on a node score compared to a cutoff (default: 0.85) (Gu and Hübschmann 2023).

### 2.5 Structural similarity and sequence similarity comparison

#### 2.5.1 Structural prediction data retrieval

UniProt accessions corresponding to the IDs of the rice genes selected based on the HN-score were retrieved using Unipressed (ver. 1.3.0) (Milton 2022) and UniProt Web Application Programming Interface (API) (accessed 23 May 2025) (UniProt Consortium 2024). Macromolecular crystallographic information files (mmCIFs) of the predicted protein structures corresponding to the UniProt accessions were retrieved using the AFDB (version 4) Web API (accessed 23 May 2025) (Varadi *et al.* 2022).

#### 2.5.2 Structural similarity search

Foldseek (ver. 9-427df8a) was used for the structural analyses (van Kempen *et al.* 2024). The AFDB database (version 4), which contains ∼214 million entries, was indexed using the foldseek databases command (Varadi *et al.* 2024). The collected rice mmCIF files were queried against the AFDB using the foldseek easy-search command. Two alignment modes were applied for structural alignment: (i) 3Di + AA Goto–Smith–Waterman local alignment (3Di + AA) and (ii) TM-alignment (Foldseek-TM), which emphasizes global structural features (van Kempen *et al.* 2024). From the resulting cross-species hits, rice–human hit pairs were extracted for downstream analysis.

#### 2.5.3 Sequence similarity

To obtain protein sequence similarity for the same set of hit pairs, the NCBI Basic Local Alignment Search Tool (BLAST) (ver.2.16.0) (Camacho *et al.* 2009) and European Molecular Biology Open Software Suite (EMBOSS) package (ver. 6.5.7) were used (Rice *et al.* 2000). All protein sequences used for structure prediction were retrieved from the AFDB FTP site (accessed 9 August 2025) (Varadi *et al.* 2024) and indexed using the makeblastdb command. Rice and human protein sequences identified via structural alignment were retrieved in the multi-FASTA format using the blastdbcmd command. The multi-FASTA files were split with EMBOSS seqretsplit, followed by pairwise alignments of rice and human protein sequences using EMBOSS Needle for global alignment and EMBOSS Water for local alignment.

#### 2.5.4 Filtering criteria

To focus on global structural similarity, minimize redundant matches, and ensure a clear gene-level analysis of human data, the following filters were applied to rice–human Foldseek hit pairs:

1. **Alignment coverage**: Coverage of 50% or more on both proteins to prioritize global over local alignments.
2. **Redundancy removal within each rice gene:** When multiple rice UniProt accessions from the same gene were aligned to the same human structure, the alignment with the highest coverage was retained, and ties were broken using the average Local Distance Difference Test (lDDT) score (Mariani *et al.* 2013).
3. **Human cross-reference**: Only hit pairs with human UniProt accessions that could be unambiguously mapped to a current Ensembl gene ID and HUGO Gene Nomenclature Committee (HGNC) gene symbol (Seal *et al.* 2023) via TogoID (accessed on 9 August 2025) (Ikeda *et al.* 2025) were retained to ensure gene-level consistency for downstream interpretation.

For the filtered hit pairs, human gene expression data under heat-stress conditions were integrated by assigning each hit pair a human HN-score (from a previous meta-analysis of public human heat stress-related transcriptome data) based on the human HGNC gene symbol (Yonezawa 2023, Yonezawa and Bono 2023).

To identify hit pairs with contrasting levels of sequence and structural similarity, the retained rice–human hit pairs were classified using two metrics: (i) global pairwise sequence similarity (%) computed by EMBOSS Needle (global alignment, default parameters) and (ii) the average lDDT score for the structural similarity metrics. Percentile thresholds (Q2 = median; Q3 = 75th percentile) were computed across all retained pairs after filtering:

• Low-sequence similarity/High-structural similarity (LS–HS): sequence similarity ≤ Q3 and average lDDT ≥ Q2.

• High-sequence similarity/High-structural similarity (HS–HS): sequence similarity > Q3 and average lDDT ≥ Q2.

• The sequence similarity cutoff at the 75th percentile (Q3) was selected to retain a sufficient number of LS–HS pairs for downstream analyses while separating high-similarity matches into HS–HS.

• Finally, “unique LS–HS” pairs were defined as LS–HS pairs with a rice gene lacking an HS–HS pair; LS–HS pairs from rice genes that had at least one HS–HS pair were excluded from the “unique” set.

### 2.6 Validation of hit pairs using other public database resources

To compare the selected structural hit pairs with existing knowledge, two types of information from public databases were integrated.

1. Domain information: For each rice and human protein, InterPro domain IDs were retrieved via UniProt (accessed 9 August 2025) (UniProt Consortium 2024, Blum *et al.* 2025) to confirm that they shared a common protein domain.
2. Orthologous relationships: Using rice gene IDs as a query, the Ensembl REST API (Comparative Genomics; Pan-taxonomic compara) (Dyer *et al.* 2025) (accessed 24 May 2025) was used to identify orthologous relationships between each rice gene and its human counterpart (API example: https://rest.ensembl.org/homology/id/oryza_sativa/Os01g0180800? compara=pan_homology&content-type=application/json; target_taxon=9606).

### 2.7 Workflow implementation

To generalize and expand the analysis process implemented in Section 2.5, the “plant2human workflow” was developed, as described in the Common Workflow Language (CWL) (Crusoe *et al.* 2022, Yonezawa 2025). This series of processes ultimately produced a Jupyter notebook as an analysis report, allowing users to refine the filtering criteria and adopt additional actions as necessary. This workflow is available for free download from the WorkflowHub website under an MIT License (Gustafsson *et al.* 2025).

## 3 Results

### 3.1 Functional annotation workflow

**Figure 1:**
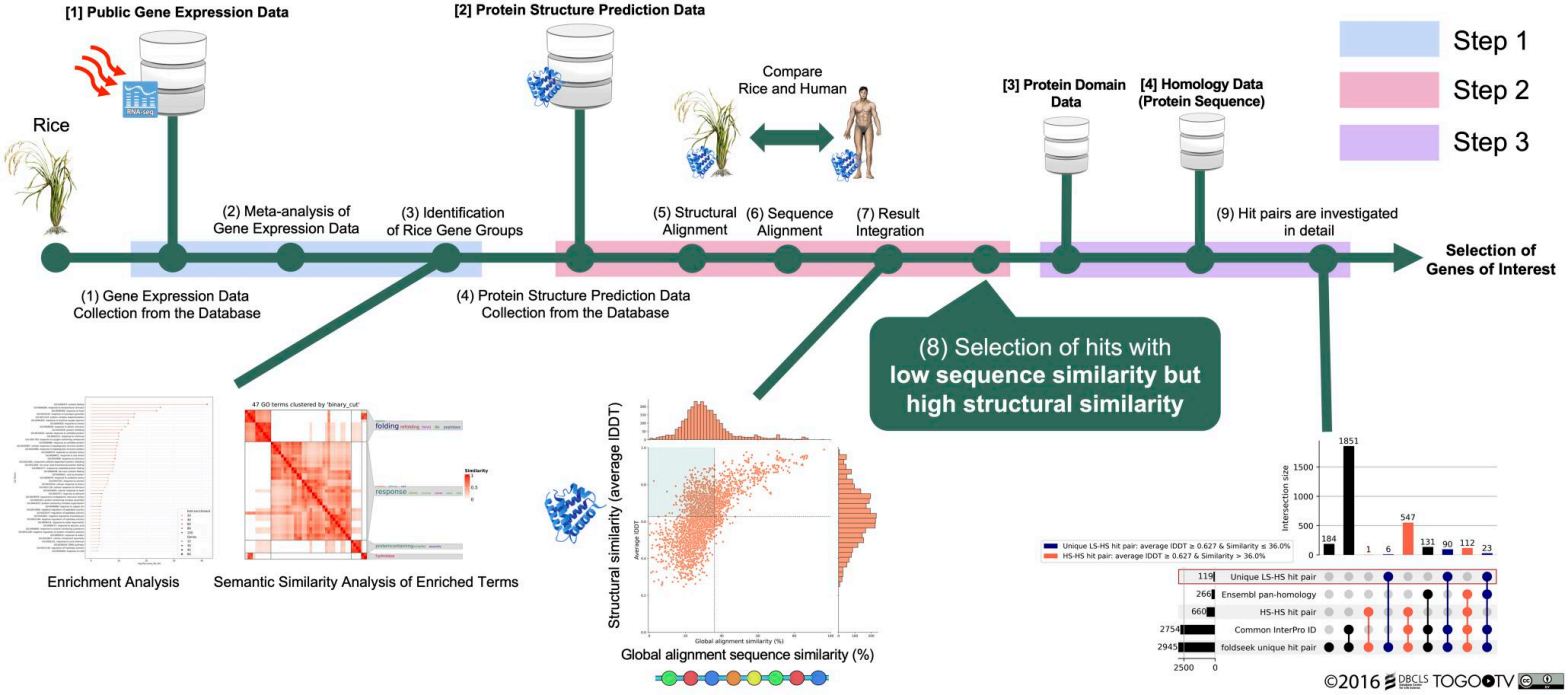
Summary of the functional annotation workflow in rice heat stress-responsive genes.

Figure 1 outlines the three-step functional annotation workflow of this study. First, a meta-analysis of rice RNA-Seq data under heat stress identified the upregulated and downregulated genes. Second, the predicted protein structures and sequences from these genes were aligned between rice and humans to identify pairs with low sequence similarity and high structural similarity (unique LS–HS condition) between rice and humans. Third, the selected hit pairs were further investigated using other public databases.

### 3.2 Gene group evaluation

We manually curated 360 pairs (heat stress vs. control conditions) of rice heat stress-related RNA-Seq data from 13 projects in the NCBI GEO (Fig. 1). The collected data comprised a wide range of experimental conditions, including heat shock and prolonged exposure to heat stress (e.g. high night temperature stress). Background information was available as a manually collected metadata CSV file (Supplementary Table S1, available as supplementary material at *Bioinformatics Advances* online) (Yonezawa and Bono 2025). Background information with complex relationships was visualized using a Sankey diagram (Supplementary Fig. S1, available as supplementary material at *Bioinformatics Advances* online) (Yonezawa and Bono 2025).

**Figure 2:**
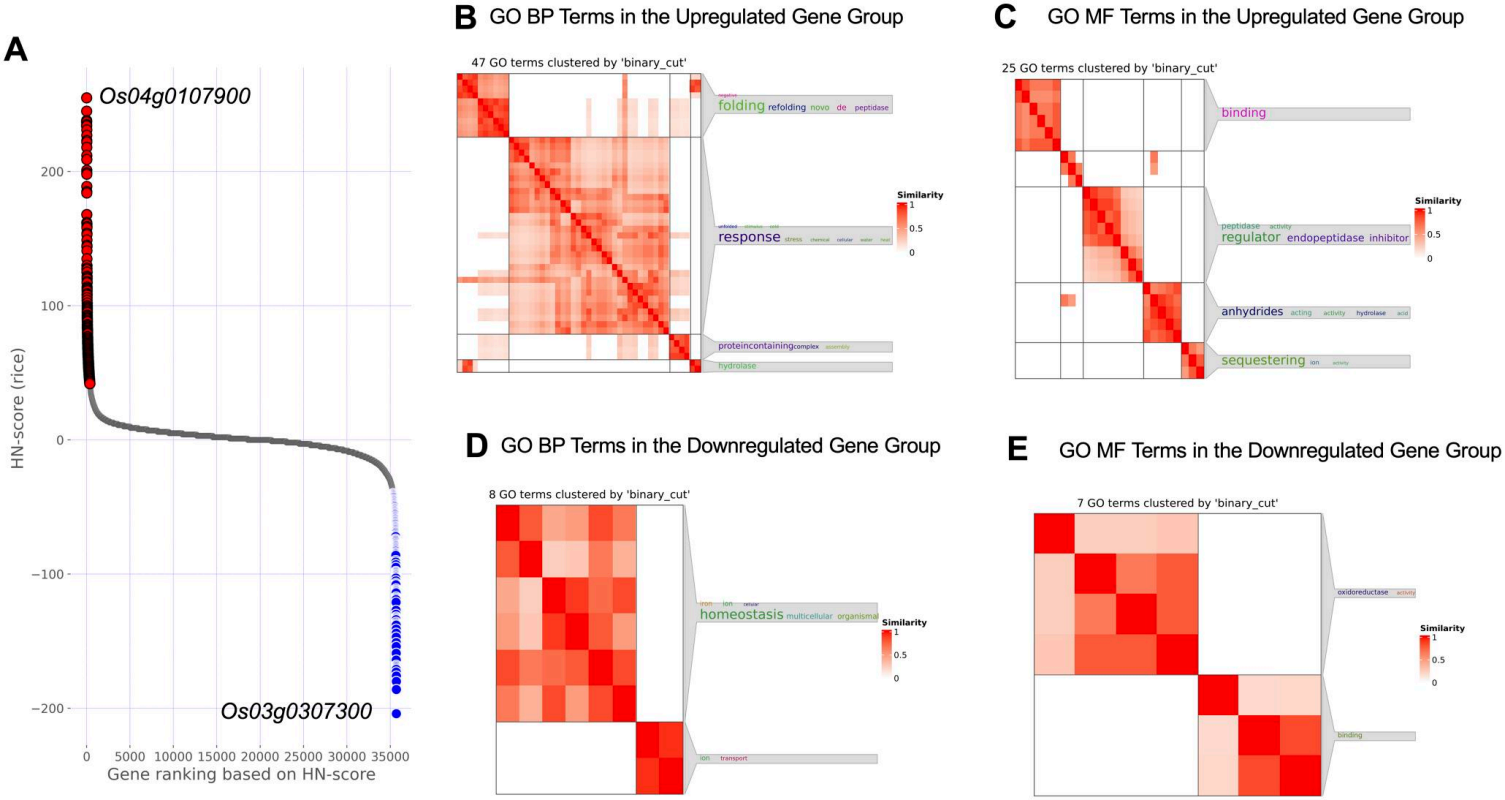
Characteristics of the gene groups selected by the HN-score. (A) Scatter plot of HN-scores for all rice genes; red: upregulated (*n *= 367), blue: downregulated (*n *= 370). Genes shown at the top left and bottom right indicate genes with the highest (255) or lowest (−204) HN-scores, respectively. (B–E) Semantic similarity heatmaps of enriched GO terms clustered by the *binary cut* algorithm (“binary_cut”) implemented in SimplifyEnrichment. Word clouds shown on the right summarize enriched keywords automatically extracted from GO term names within each cluster; Fisher’s exact test assesses keyword enrichment, and the significance is mapped to keyword font size (Gu and Hübschmann 2023). Accordingly, multi-word phrases can appear as separate words (e.g. “de” and “novo” from “de novo”). (B, C) Clustering results of GO terms enriched in the upregulated group: (B) BP; (C) MF. (D, E) Clustering results of GO terms enriched in the downregulated group: (D) BP, (E) MF. CC clusters are shown in Supplementary Fig. S3, available as supplementary material at *Bioinformatics Advances* online (Yonezawa and Bono 2025). Abbreviations: BP, biological process; CC, cellular component; MF, molecular function.

Subsequently, expression quantification, HN-ratio calculation (expression ratio), and evaluation index (HN-score) calculation were performed on the collected rice RNA-Seq data (Supplementary Tables S2–4, available as supplementary material at *Bioinformatics Advances* online) (Yonezawa and Bono 2025). Based on the HN-score, rice genes within the top 1% (42 ≤ HN-score ≤ 255; 367 genes) and bottom 1% (−204 ≤ HN-score ≤ −40; 370 genes) were selected as the upregulated and downregulated gene groups, respectively (Fig. 2A; Supplementary Table S5, available as supplementary material at *Bioinformatics Advances* online) (Yonezawa and Bono 2025).

GO enrichment analyses (Supplementary Fig. S2 and Supplementary Table S6, available as supplementary material at *Bioinformatics Advances* online) (Yonezawa and Bono 2025) were performed to characterize both groups, and the enriched terms were clustered based on semantic similarity (calculated using simona; Gu 2024) using the *binary cut* algorithm implemented in SimplifyEnrichment (Gu and Hübschmann 2023) (Fig. 2B–E; Supplementary Fig. S3, available as supplementary material at *Bioinformatics Advances* online) (Yonezawa and Bono 2025). The upregulated gene group included GO: 0009408, “response to heat” (Fig. 2B), and GO: 0051082, “unfolded protein binding” clusters (Fig. 2C), aligning with our Step 1 curation and HN-score ranking by effectively capturing heat stress-responsive genes. In the downregulated gene group, a cluster related to homeostasis (e.g. GO: 0098771, inorganic ion homeostasis; Fig. 2D) was detected, as well as a separate cluster including GO: 0016491 oxidoreductase activity (Fig. 2E). Clusters associated with metal ion-related metabolism—especially iron—were observed: the upregulated group included GO: 0140315 iron ion sequestering activity (such as Os11g0106700, Os12g0106000, and Os09g0396900), whereas the downregulated group included GO: 0006826 iron ion transport (such as Os02g0649900) (Fig. 2C and D). Furthermore, the term enriched in the upregulated gene group included GO: 0046688 response to copper ion (Os03g0266900, Os03g0267000, and Os03g0267200) (Supplementary Fig. S2 and Supplementary Table S6, available as supplementary material at *Bioinformatics Advances* online) (Yonezawa and Bono 2025).

### 3.3 Structurome analysis

**Figure 3:**
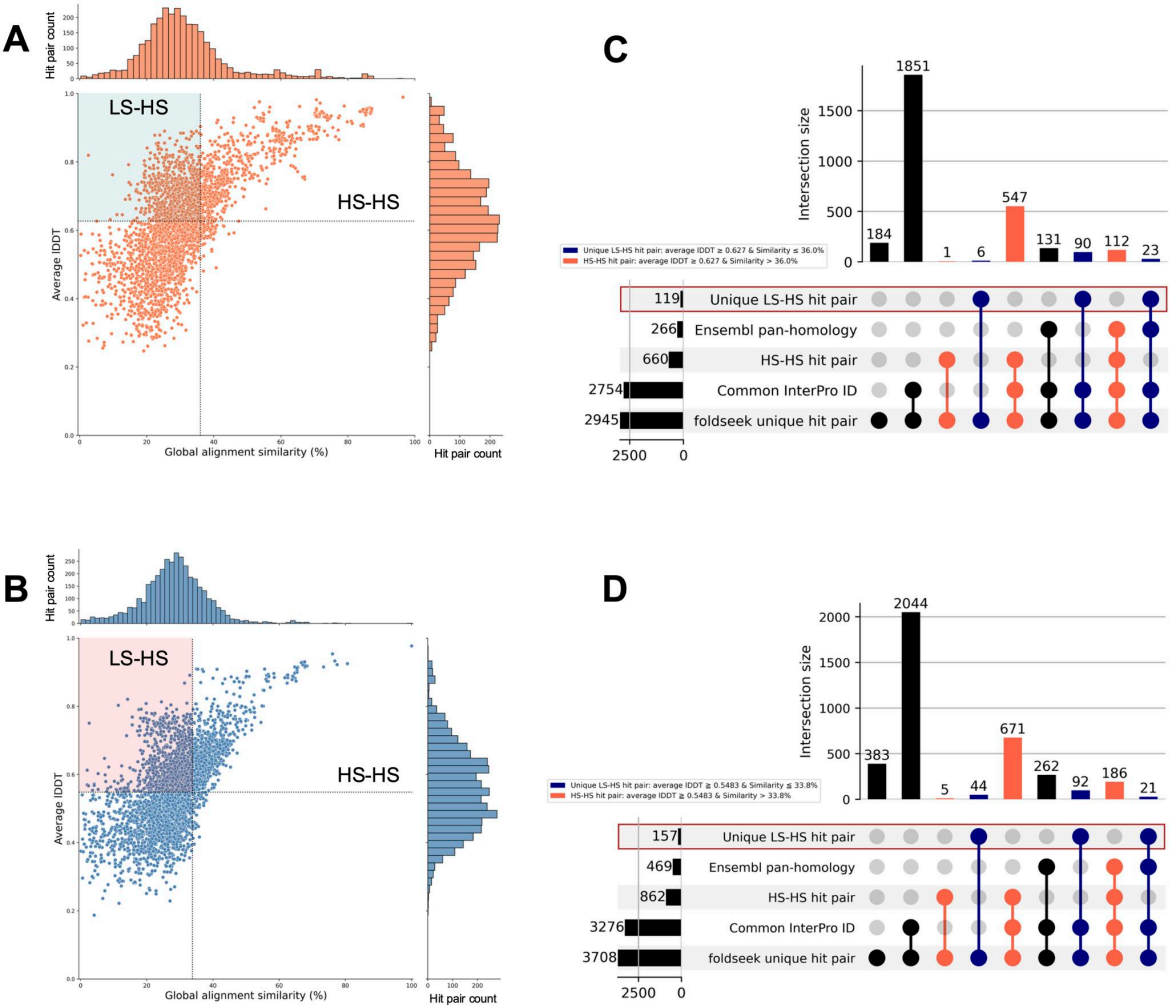
Landscape of rice–human structural/sequence relationships and characterization of hit pairs using public database information. (A, B) Scatter plots of average lDDT (y-axis) versus global pairwise sequence similarity (%) computed using EMBOSS Needle (x-axis) for the retained rice–human pairs. Vertical and horizontal lines denote Q3 (sequence similarity) and Q2 (average lDDT), respectively. (A) Derived from the upregulated gene list. Vertical line Q3 = 36.0%; horizontal line Q2 = 0.627; (B) derived from the downregulated gene list. Vertical line Q3 = 33.8%; horizontal line Q2 = 0.5483. (C, D) UpSet plots showing intersections among five attributes based on hit pair count. Rows (bottom to top): Foldseek all unique hit pairs, Common InterPro ID (share at least one InterPro ID), HS–HS hit pair (sequence similarity > Q3, average lDDT ≥ Q2), Ensembl pan-homology (ortholog relationships based on protein sequence), and Unique LS–HS hit pair (sequence similarity ≤ Q3, average lDDT ≥ Q2). Left horizontal bars indicate the set size of each row. Columns indicate set intersections; closed circles denote membership, and vertical connectors denote logic. The bar height at the top shows the intersection size (number of hit pairs); intersections are categorized as those consisting exclusively of “Unique LS–HS hit pair”, those consisting exclusively of “HS–HS hit pair”, and all other intersections. (C) upregulated; (D) downregulated.

Using the gene lists described in Section 3.2, the predicted rice protein structures were compared with those of humans, focusing on LS–HS candidates by combining structural and sequence information (Step 2 in Fig. 1). Pairwise sequence alignment results, together with structural alignment data, were used to identify protein hit pairs across distantly related species. Predicted structures were obtained from the AFDB (version 4), and structural searches were conducted using Foldseek (3Di + AA and Foldseek-TM method) against the complete AFDB database (van Kempen *et al.* 2024, Varadi *et al.* 2024); rice–human hits were retained (Supplementary Table S7, available as supplementary material at *Bioinformatics Advances* online) (Yonezawa and Bono 2025). The 3Di + AA results served as the primary analysis; the Foldseek-TM findings are summarized in Supplementary Fig. S4 and Supplementary Table S8, available as supplementary material at *Bioinformatics Advances* online (Yonezawa and Bono 2025). After pairwise sequence alignment and the application of predefined filters, 2945 hit pairs were identified in the upregulated gene group (145/367 genes) and 3708 hit pairs in the downregulated gene group (147/370 genes). The distributions of the average lDDT and global sequence similarity (%) are shown in Fig. 3A and B, with additional metrics and indicators in Supplementary Fig. S5, available as supplementary material at *Bioinformatics Advances* online, and all hit pairs in Supplementary Table S9, available as supplementary material at *Bioinformatics Advances* online (Yonezawa and Bono 2025).

Within the LS–HS regions (Fig. 3A and B), 119 hit pairs were identified (38/367 genes) in the upregulated gene group and 157 hit pairs (34/370 genes) in the downregulated gene group after excluding rice genes that also had HS–HS matches. We defined these as “unique LS-HS” hit pairs (for details, see Section 2, Section 2.5.4).

To further characterize these hits, we compared “unique LS–HS” and HS–HS pairs using InterPro domain information and ortholog information between rice and humans retrieved from Ensembl (Fig. 3C and D; Step 3 in Fig. 1) (Yates *et al.* 2022, Blum *et al.* 2025). By combining these two types of information, we identified hit pairs in which existing data (domain and ortholog information or domain information alone) supported the correspondence of rice–human structural hit pairs or cases in which both types of information were missing (Fig. 3C and D).

We next examined whether the human counterparts of these unique LS–HS pairs exhibit transcriptional heat responses using publicly available meta-analysis of heat stress-related transcriptomes in humans quantified by HN-score (human HN-score) (Yonezawa 2023, Yonezawa and Bono 2023). The human HN-score used in the previous analysis was calculated using the same HN-ratio (five-fold, 1/5-fold) as in this study. At the level of whole unique LS–HS hit-pair, 11 of 119 hit pairs derived from the rice upregulated gene group mapped to human upregulated genes (top 500 genes by human HN-score), whereas 1 of 157 hit pairs derived from the rice downregulated gene group mapped to human downregulated genes (bottom 500 genes by human HN-score) (for more details, see Supplementary Table S9, available as supplementary material at *Bioinformatics Advances* online) (Yonezawa and Bono 2025).

To demonstrate specific examples, six representative rice–human unique LS–HS hit pairs were selected (Table 1) based on the following criteria. These six examples were selected, with three genes from each group: the upregulated and downregulated gene groups. First, hit pairs were identified across three evidence categories defined by external annotations: pairs supported by both common InterPro domains and Ensembl pan-homology, pairs supported only by common InterPro domains, and pairs with neither type of support. Second, AFDB entries corresponding to the UniProt accessions shown in Table 1 were confirmed to remain available in AFDB version 6 (synchronized with UniProt release 2025_03) (AlphaFold Database Release Notes 2025). AFDB version 6 was released after our analyses were performed; therefore, the primary analyses were not re-run with version 6. Third, the selected rice genes were not detected in the enrichment analysis (Supplementary Fig. S2 and Table S6, available as supplementary material at *Bioinformatics Advances* online) (Yonezawa and Bono 2025). For each selected hit pair, two interpretability flags were also reported: whether the pair was recovered using an alternative structural alignment mode (Foldseek-TM, concordant) and whether the rice protein matched multiple human structures (which may require caution in interpretation). All hit pairs and details of unique LS–HS and HS–HS interactions are presented in Supplementary Table S9, available as supplementary material at *Bioinformatics Advances* online (Yonezawa and Bono 2025).

**Table 1:** Representative unique LS–HS rice–human hit pairs.

Rice gene ID	HN-score	Rice UniProt accession	Human gene symbol^a^	Human UniProt accession	Common InterPro ID^b^	Ensembl Pan-homology^c^
*Os08g0442200*	48	A0A0P0XGD7	*APMAP*	Q9HDC9	Yes	Yes
*Os09g0444900*	45	A0A0P0XNJ7	*TMEM45A*	Q9NWC5	Yes	No
*Os03g0817100* ^d^	54	B9F6Z0	*MAL* ^e^	P21145	No	No
*Os07g0190000*	−64	Q6YU51	*TKT* ^e^	P29401	Yes	Yes
*Os03g0372600* ^d^	−46	Q84TS2	*ATOX1* ^e^	O00244	Yes	No
*Os07g0524200* ^d^	−72	Q69SA8	*TMEM106A*	K7ERE2	No	No

a Human gene symbol: HGNC gene symbols obtained using TogoID (9 August 2025, date last accessed).

b Common InterPro ID: ≥1 shared InterPro ID retrieved via the UniProt (9 August 2025, date last accessed).

c Ensembl Pan-homology: any human ortholog correspondence reported by Ensembl pan-taxonomic Compara (24 May 2025, date last accessed).

d Concordant hit pair: Concordant indicates that the same rice–human hit pair was identified as an LS–HS hit pair in both analyses, i.e. when LS–HS filtering was applied separately to the results of the primary 3Di + AA mode and the alternative Foldseek-TM mode (each with its own threshold).

e Multiple hit pair: This indicates that the corresponding rice protein matched multiple human AFDB structures (derived from different gene products); the human gene symbol shown in Table 1 is one of the multiple human hits. The top-ranked hit (highest average lDDT) is reported in the table.

## 4 Discussion

This study presents an analytical workflow integrating public transcriptome and structural data to investigate heat stress response genes in rice.

### 4.1 Heat stress response system and metal metabolism

Micronutrient metals such as iron and copper are essential for the survival of land plants, including rice. The molecular mechanisms responsible for regulating metal homeostasis and the associated genes are well characterized (Krämer 2024). Through a meta-analysis of publicly available transcriptome data under heat stress conditions and accompanying structural analysis, genes related to metal homeostasis were identified.

Enrichment analysis with Gene Ontology (GO) and semantic similarity analysis revealed that genes involved in iron ion sequestration were upregulated, whereas those involved in iron ion transport were downregulated in rice (Supplementary Table S6, available as supplementary material at *Bioinformatics Advances* online) (Yonezawa and Bono 2025; Fig. 2C and D). These changes may reflect characteristics observed in ferroptosis under heat stress. The upregulated gene set included *Os12g0106000* (gene symbol: *OsFER2*), which positively regulates ferroptotic cell death when rice is infected with avirulent *Magnaporthe oryzae* as a form of biotic stress (Nguyen *et al.* 2022). Furthermore, in *A. thaliana*, regulated cell death under heat stress occurs in the form of ferroptosis-like cell death (Distéfano *et al.* 2017). However, direct evidence explaining the coordinated regulation of these gene sets under heat stress remains limited. Recent research has explored the interaction between temperature stress and iron. For instance, studies on light-chilling stress in cucumbers (*Cucumis sativus L.*) demonstrate that high-iron nutritional conditions can exacerbate oxidative damage via light-dependent root-to-shoot iron translocation and lipid peroxidation, whereas low-iron conditions may mitigate these effects (Takeuchi *et al.* 2024). As such, insights into iron nutrition management have direct implications for agricultural applications (Takeuchi *et al.* 2024).

Enrichment and structural analyses in humans identified genes potentially related to copper and iron metabolism. The upregulated gene group was enriched for the GO: 0046688 response to copper ion (Supplementary Fig. S2 and Supplementary Table S6, available as supplementary material at *Bioinformatics Advances* online) (Yonezawa and Bono 2025). Structural comparisons with humans highlighted gene pairs like *Os03g0372600–ATOX1* (Table 1, row 5), sharing the heavy metal-associated InterPro domain (IPR006121, accessed 21 August 2025). Although a rice gene belonging to the same domain (IPR006121), *Os02g0196600* (*OsHMA4*), is known to transport copper to root vacuoles (Huang *et al.* 2016), *Os03g0372600* remains understudied {*LOC107276793 Heavy Metal-Associated Isoprenylated Plant Protein 43 [Oryza Sativa Japonica Group (Japanese Rice)]—Gene—NCBI* 2024}. Insights from human copper counterparts—*ATOX1* (antioxidant 1 copper chaperone)—and the emerging concept of cuproptosis—copper-dependent cell death (Chen *et al.* 2025)—can inform hypothesis generation for heat-stress response systems in rice.

High temperatures affect rice throughout its life cycle and across various plant organs. Genes linked to heat tolerance have been associated with plant growth stages, organs, and thermophenotypes (Ren *et al.* 2023). Further investigation into metal metabolism-related genes identified in this study, particularly their thermophenotypes, could aid in future agricultural applications.

### 4.2 Interpretation of representative unique LS–HS hit pairs

Besides the *Os03g0372600*–*ATOX1* pair, our screening identified many rice–human unique LS-HS hit pairs. To illustrate how such hit pairs can be interpreted, we present six representative hit pairs in Table 1, including *Os03g0372600–ATOX1*. These examples were selected to demonstrate how external annotations can facilitate interpretation of structure-based hits; therefore, we categorize the six pairs into three evidence tiers based on common InterPro domains and Ensembl pan-homology information.

Pairs supported by both common InterPro domains and Ensembl pan-homology [e.g. *Os08g0442200–APMAP* (adipocyte plasma membrane associated protein), Table 1, row 1; *Os07g0190000–TKT* (transketolase), Table 1, row 4] represent strongly supported candidates. In such cases, domain information provides a broad functional context, while Enssembl pan-homology information explicitly indicates a correspondence consistent with the structure-based relationship, thereby narrowing down candidates within a domain-level group and enhancing prioritization for targeted functional follow-up and cross-species annotation transfer. For example, *APMAP* (previous symbols: *C20orf3*) exhibits arylesterase activity (Ilhan *et al.* 2008), while *TKT* is an enzyme in the pentose phosphate pathway (Boyle *et al.* 2016). These findings in humans suggest that specific function prediction, such as substrate prediction, is possible, provided these data are carefully validated.

Pairs supported by common InterPro domains but lacking Ensembl pan-homology [e.g. *Os09g0444900-TMEM45A* (transmembrane protein 45A), Table 1, row 2; *Os03g0372600*–*ATOX1* (Table 1, row 5)] remain interpretable candidates, because conserved domain architecture can provide functional context. For example, the *Os09g0444900–TMEM45A* pair shares the InterPro entry IPR006904 (Protein of unknown function DUF716). Although this domain is uncharacterized, the common domain annotation provides an interpretable anchor that, together with structural similarity, motivates further investigation of the rice gene. Interestingly, *TMEM45A* is upregulated under hypoxic conditions and has been suggested to play a crucial role in hypoxia-induced chemoresistance (Flamant *et al.* 2012). This is also indicated in a meta-analysis of hypoxic human transcriptome data (Ono 2021, Ono and BOno 2021). Thus, it is possible to hypothesize that *Os09g0444900*, which shares a similar structure with a common InterPro domain, may be involved in a stress response.

Finally, pairs lacking both common InterPro domains and Ensembl pan-homology {e.g. *Os03g0817100–MAL* [mal, T cell differentiation protein (MAL blood group)], Table 1 row 3; *Os07g0524200–TMEM106A* (transmembrane protein 106A), Table 1 row 6} represent structure-driven candidates. These results should be interpreted with caution, but these hit pairs might indicate incomplete sequence annotation at this time, making it especially helpful for generating hypotheses. For example, as demonstrated by the verification of the ortholog of human *BRCA2* in *Arabidopsis* (Siaud *et al.* 2004), this could provide an opportunity to investigate whether phenotypes observed in humans are also observed in rice.

In addition to interpretation through integration with InterPro domain information and Ensembl pan-homology information, we conducted a simplified investigation using human HN-scores to determine whether the unique LS-HS hit pairs identified in this study exhibit similar gene expression patterns in humans as reported in a previous study (Yonezawa 2023, Yonezawa and Bono 2023). None of the six representative unique LS–HS hit pairs involved human genes previously classified into the human upregulated/downregulated gene group (top/bottom 500 genes by human HN-score) (Supplementary Table S9, available as supplementary material at *Bioinformatics Advances* online) (Yonezawa and Bono 2025). This does not suggest that the approach is incorrect; rather, it shows that structure-based mapping can yield functional hypotheses that complement gene-expression-based screening.

Overall, in addition to six representative examples, the unique LS-HS hit pairs identified in this study can be integrated with resources and datasets from other public databases for interpretation, facilitating hypothesis generation.

### 4.3 Comparison with distantly related species through structural similarity

As a proof-of-concept, we aimed to identify novel heat stress-responsive genes using public transcriptomes and structures. Our selection of humans as the target organism was motivated by the opportunity to utilize conserved protein structural features, rather than strict orthology, to map rice proteins into a comprehensively annotated human context (Illergård *et al.* 2009). For instance, hit pairs with high structural similarity but uneven annotations, such as *Os03g0372600*–*ATOX1* pair (Table 1, row 5), enable the transfer of human annotations to rice, generating testable hypotheses.

Additionally, our findings suggest that conserved protein structure can support the use of rice as a model organism for humans—a concept consistent with prior validations of *BRCA2* in *Arabidopsis* and confirmation of human disease gene orthologs in *Arabidopsis* and *P. trichocarpa* (Wang *et al.* 2023, Siaud *et al.* 2004). With resources like the AFDB now offering large-scale protein structure datasets (Varadi *et al.* 2024), leveraging conserved protein structures presents a scalable approach for nominating a broader range of species as hypothesis-generating model organisms relevant to human research. For example, metal metabolism highlighted in Section 4.1 underscores the translational potential of plant findings for human biology. Such comparative analyses are facilitated by the “plant2human workflow,” implemented in CWL (Crusoe et al. 2022, Yonezawa 2025), which allows systemic mapping between genes in various species and their human counterparts via the structurome. This workflow enhances biological discovery by enabling reciprocal functional annotation across species.

To investigate distant functional protein hit pairs that may not be detected by sequence alignment-based methods, low (global) sequence similarity combined with high structural similarity (LS–HS condition) was used in conjunction with three filtering conditions. This methodology draws upon the concept of the “twilight zone,” a region in sequence alignment where homology inference is difficult due to sequence identity limitations (Rost 1999). Recent approaches targeting proteins in the twilight zone use physicochemical property-based methods (Dixson and Azad 2025). In this study, the approach was extended by introducing an exploratory “user-defined twilight zone” referred to as the LS–HS condition to facilitate the detection of understudied genes. Sequence similarity served as a general indicator rather than an optimization target for structural hit pairs; thus, sequence-alignment parameters were maintained at default values (see Section 2.5.3). Although further refinement could enhance the method, it is likely to contribute new perspectives in evaluating individual genes, including those excluded from enrichment analyses.

However, continuous updating of the sequence information recorded in UniProt could lead to hit pairs with discrepancies between structural and domain information. Therefore, there are hit pairs that require actions, such as reacquiring domain information and using AlphaSync, which synchronizes protein structure prediction data (Lang *et al.* 2025).

### 4.4 Limitation

This study has several methodological limitations that could lead to misannotation. First, we did not impose stringent quality-based filtering on AlphaFold predictions [e.g. predicted Local Distance Difference Test (pLDDT) thresholds (Varadi *et al.* 2022)], prioritizing coverage to consider more genes. This choice increases sensitivity but may also lead to inflated false-positive structural matches, especially in regions of low confidence. Second, our comparison focused on more global structural similarities and did not evaluate detailed structural verification, such as that performed for the rice HSP90 protein structure (Raman and Suguna 2015). Therefore, interpreting gene functions still requires careful consideration, necessitating experimental verification using technologies such as genome editing, as in previous studies that identified unknome (Rocha *et al.* 2023). Third, regarding generalizability, while the core framework of the plant2human workflow is, in principle, extensible to other species pairs with predicted structures available in AFDB, the current implementation and analysis in this article focus on the rice-human setting. This is because our downstream interpretation step is designed around unambiguous mapping to HGNC gene symbols, which serve as a hub for integrating diverse human annotation resources (Section 2.5.4). However, comparisons with other species (e.g. mouse) may also yield novel biological insights that cannot be obtained solely from comparisons with humans. Thus, we plan to continue updating the plant2human workflow and expand the supported target species.

## 5 Conclusion

This study focuses on poorly understood heat stress-responsive genes in rice by integrating resources from public life science databases, particularly public transcriptome and structural data. This approach identifies understudied genes and suggests the potential for functional annotation using information from other species, such as humans, through structurome analysis. Furthermore, the newly developed plant2human workflow is effective for heat stress and exploring gene groups with diverse biological backgrounds.

## Supplementary Material

vbag013_Supplementary_Data

## Data Availability

All codes in this study are publicly available at https://github.com/yonesora56/HS_rice_analysis and are licensed under the MIT license. This analysis workflow, executed in Sections 2–5, is available as a “plant2human workflow” on WorkflowHub, licensed under the MIT license (Gustafsson *et al.* 2025, Yonezawa 2025).
