# Supplementary material for: Functional annotation of novel heat stress-responsive genes in rice utilizing public transcriptomes and structurome
Source: Bioinform Adv. 2026 Jan 21;6(1):vbag013. doi: 10.1093/bioadv/vbag013 (PMC12889164; doi:10.1093/bioadv/vbag013)
Supplement: vbag013_Supplementary_Data [file vbag013_supplementary_data.zip › publicweb_zipdownload.go]

Proself
